# An indolent case of isolated cerebral mucormycosis: an uncommon presentation

**DOI:** 10.1099/acmi.0.000023

**Published:** 2019-05-07

**Authors:** David J. Montgomery, Randi S. Goldstein, Dontre' M. Douse, Jerome Tuitt, Michael Sinnott

**Affiliations:** ^1^​ Mercer University School of Medicine, Georgia, USA; ^2^​ Memorial Health University Medical Center, Department of Internal Medicine, Savannah, Georgia, USA; ^3^​ Memorial Health University Medical Center, Department of Pathology, Savannah, Georgia, USA

**Keywords:** intracranial mucormycosis, intracerebral mucormycosis, mucormycosis, isolated mucormycosis, atypical mucormycosis presentation

## Abstract

**Introduction:**

This case is a presentation of isolated central nervous system (CNS) Mucormycosis in an immunocompetent patient. This case is unique in its demonstration of isolated CNS involvement while lacking clear evidence elucidating an entry point.

**Case presentation:**

The patient is a 36-year-old man without a pertinent past medical history, who initially presented with altered mental status and a 5-day history of progressively slurred speech. His social history is significant for intravenous drug use and outdoor pest control work. The patient’s head computed tomography (CT) scan without contrast demonstrated the presence of possible bilateral infarcts or masses involving the basal ganglia and periventricular white matter. The patient then progressed to facial diplegia with new onset hemiplegia. High-dose steroids were initiated due to concern for neurosarcoidosis. A lumbar puncture was ordered due to minimal improvement and suggested an inflammatory process. A stereotactic brain biopsy was then performed, demonstrating non-caseating granulomatous inflammation with giant cells. Liposomal amphotericin B was added to cover possible fungal etiology. The pathology report was consistent with an isolated cerebral mucormycosis infection. The etiology remained elusive with clear paranasal sinuses and no cutaneous manifestations. Due to extensive gray matter involvement, the patient was not a candidate for surgery.

**Conclusion:**

This is a report of mucormycosis in a seemingly immunocompetent patient with either isolated CNS involvement or disseminated mucormycosis without an identifiable source. Although this patient did have two risk factors including intravenous drug use and outdoor working history, his lack of peripheral involvement demonstrates an uncommon presentation.

## Introduction

This case is a presentation of isolated CNS mucormycosis in an immunocompetent patient. Mucormycosis is usually caused by the presence of a species from any number of the following pathogenic genera: Mucor, Rhizopus, Rhizomucor or Lichtheimia. These fungi are often found in the soil in high humidity climates with transmission occurring through exposure to spores entering either through inhalation or absorption through the skin or mucous membranes. Frequently these infections are associated with immunocompromised patients ailing from a monocyte or granulocyte deficiency or in the setting of Diabetic Ketoacidosis. This case is unique in that the only sign of infection was isolated CNS involvement in the absence of an obvious entry point, including no obvious sinus or cutaneous involvement [1, 2].

## Case Report

The patient, a 36-year-old Caucasian man, initially presented with altered mental status and a 5-day history of progressively slurred speech. The patient progressively became non-verbal and then developed fecal and urinary incontinence with watery diarrhea. This patient had no history of prior cerebral nervous system events or head trauma. In addition, there is no history of recent illnesses or any prior medical conditions. Pertinent social history includes an occupation focusing on outdoor pest and termite control for 13 years as well as an 8-year history of IV oxycodone use. There is no pertinent family history and the patient denies any outdoor hobbies. On physical exam, the patient remained afebrile, had delayed speech and communicated mainly through hand gestures. Comprehension was limited to single-step commands but strength was 5/5 bilaterally in both the upper and lower extremities. The remainder of the physical exam was unremarkable. The only laboratory abnormality discovered was a mild leukocytosis at 12 800 ul^−1^. His urine drug screen was negative, and both the patient and family denied any drug use. In addition, the urinalysis, chest x-ray and electrocardiogram were all unremarkable. The patient’s head computed tomography without contrast demonstrated the presence of possible bilateral infarcts or masses involving the basal ganglia and periventricular white matter greater on the right.

The next day neurology examined the patient and aside from new onset right facial diplegia, the physical exam findings remained unchanged from above. At that time, a magnetic resonance imaging (MRI) scan with and without contrast, a magnetic resonance angiography (MRA) scan of the head, a carotid ultrasound and an electroencephalogram (EEG) were all ordered. The EEG returned a finding of frontal intermittent rhythmic delta activity, a non-specific finding, often found in the setting of toxic or metabolic encephalopathies. The MRI results remained uncertain of whether the patient was presenting with bilateral masses or infarcts. Of note, paranasal sinuses and mastoid air cells were reportedly clear. The patient’s condition and prognosis at this time remained unclear and after several days his condition seemed to improve clinically according to family members. The patient’s improvement, perceived low suspicion for infection, and the current prevailing diagnosis of neurosarcoidosis prompted the initiation of a 5-day course of intravenous methylprednisolone 1 g/day. On day 3 of hospitalization, the patient continued to have diarrhea and reported a headache of unknown duration. Steroid administration led to a mild improvement in the patient’s condition, but the patient continued to remain mostly non-verbal.

**Fig. 1.**
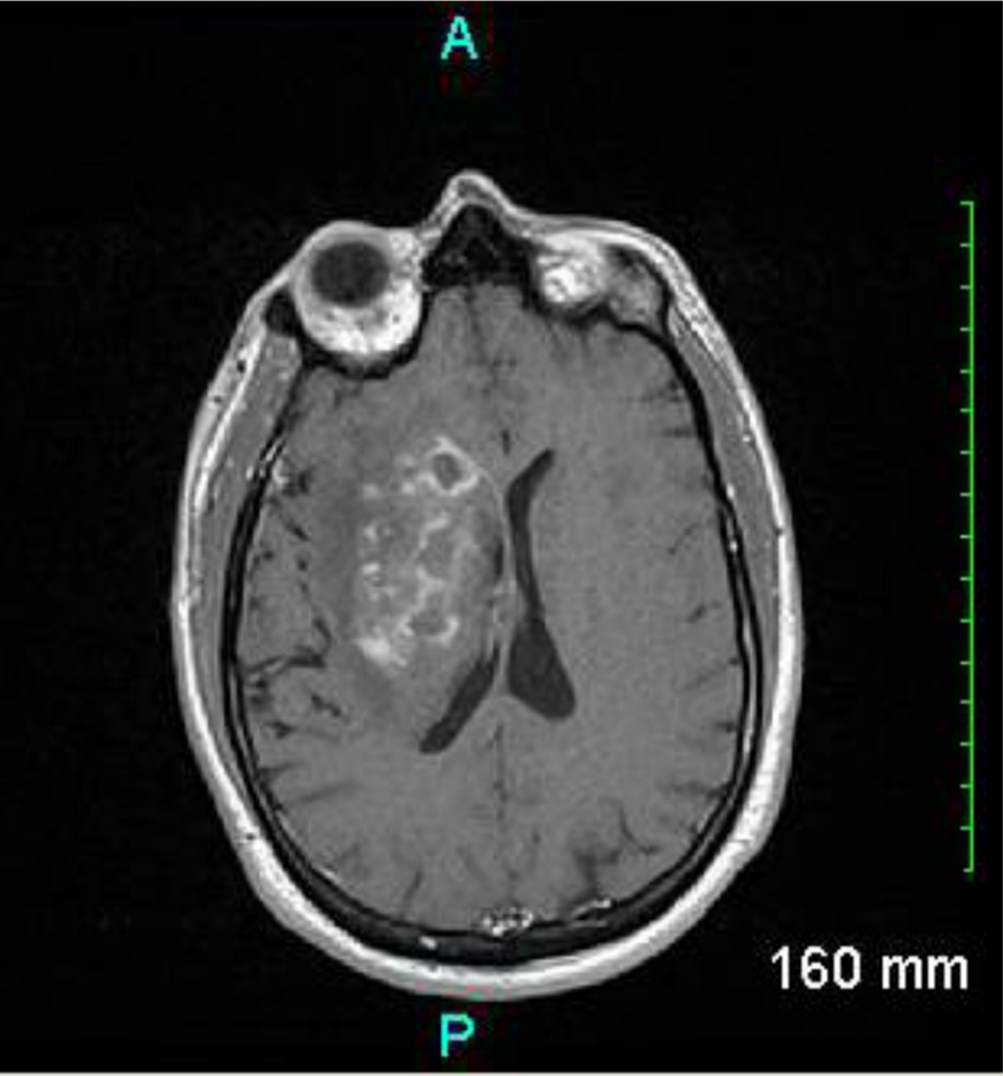
MRI of the brain with and without Omniscan IV contrast showing a heterogeneous enhancing lesion within the right basal ganglia with associated edema and local mass effect.

**Fig. 2.**
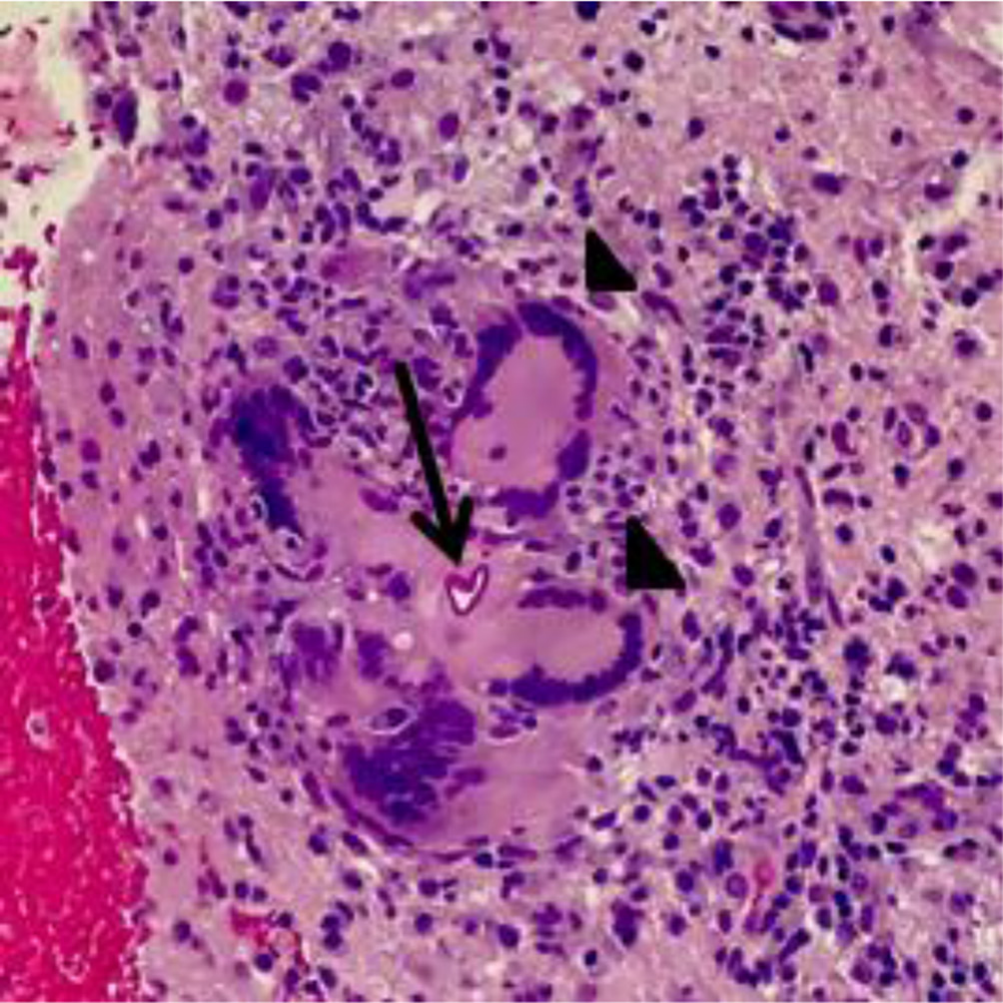
Non-necrotizing giant cells (arrow heads) surrounding fungal hyphae (black arrow). H and E stain.

**Fig. 3.**
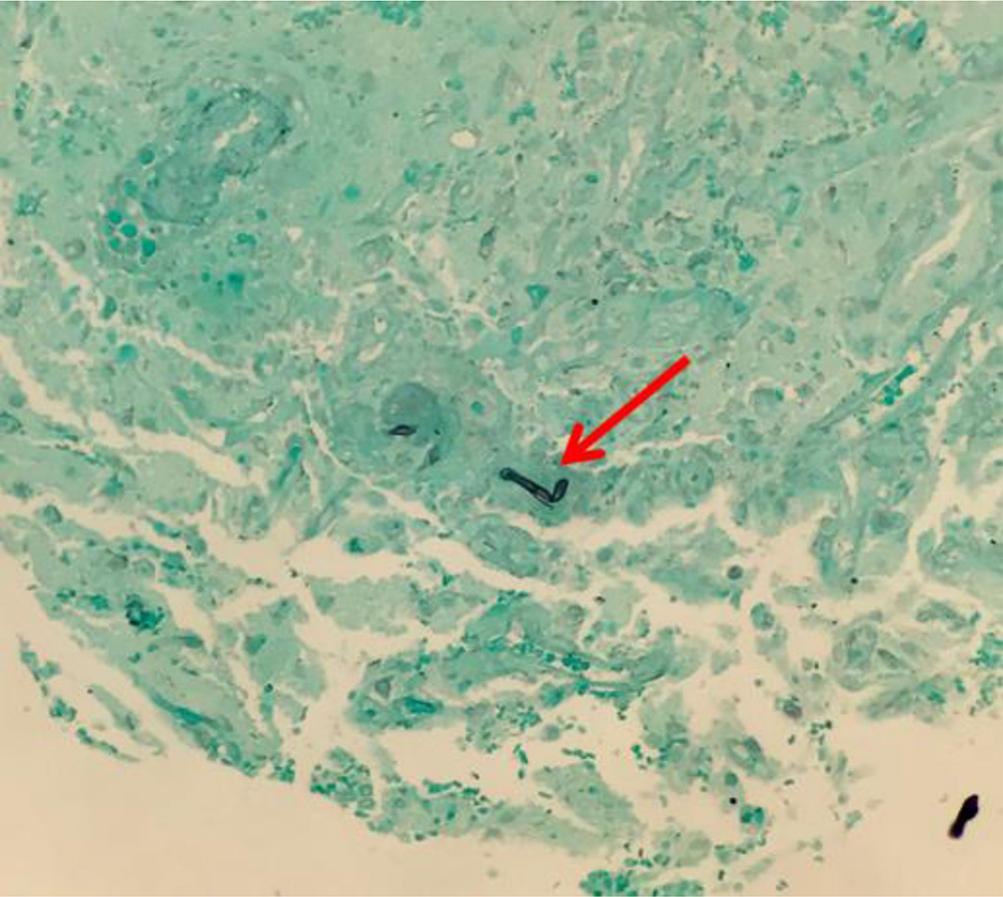
GMS special stain shows fungal hyphae, favouring non-septate/pauciseptate hyphae.

On day 6 of hospitalization, the patient developed a new onset left hemiparesis, which prompted neurology to conduct a lumbar puncture showing a red blood cell (RBC) count of 35 µl^−1^, white blood cell (WBC) count of 590 µl^−1^ with 82 % lymphocytes, a glucose of 41 mg dl^−1^, and protein of 140 mg dl^−1^. The lumbar puncture was suggestive of an inflammatory process consistent with autoimmune, paraneoplastic or viral process. This was consistent with the initial course of treatment, however due to the lack of improvement, the etiology of this neurocognitive dysfunction was reassessed. On day 7 of hospitalization, the patient had a repeat MRI that demonstrated increased cerebral edema on the right side with the center of the mass demonstrating necrosis (see Fig. 1). On day 8, the patient’s mental condition declined, his left hemiparesis worsened and the patient was no longer able to follow simple commands. Because of the prevailing thought of an autoimmune etiology, the patient was begun on dexamethasone 4 mg every 6 h. On hospitalization day 11, with recommendation from neurosurgery, the patient received a right basal ganglia and deep white matter stereotactic brain biopsy, which demonstrated non-caseating granulomatous inflammation with giant cells (see Fig. 2). With this biopsy result, the infectious disease specialist recommended the addition of liposomal amphotericin B to cover the possibility of a fungal etiology. On day 14, the full pathology report returned with Gomori methenamine-silver stain revealing the presence of fungal hyphae favoring non-septate/pauciseptate hyphae (see Fig. 3), which was consistent with an isolated cerebral mucormycosis infection. More specifically, PCR demonstrated Rhizopus oryzae fungus. Otolaryngology was consulted to identify a source of the mucormycosis infection; however, no point of entry could be ascertained as the paranasal sinuses were clear and no cutaneous manifestations were present. Due to the extent of the neurologic involvement, posaconazole was incorporated into treatment at the suggestion of the otolaryngologist. Of note, the otolaryngologist identified an otitis media but denied it as the source of the mucormycosis infection. After about 4 months of consistent antifungal therapy, the patient has improved significantly. He has regained much of his neurological functioning, including the ability to speak intelligibly and purposefully move his extremities. While the patient is still in recovery, he is slowly approaching a level commensurate with his pre-infection state.

## Discussion

This case describes an unusual case of isolated intracranial mucormycosis. The literature dating back to 1982 lists certain risk factors associated with mucormycosis infection. Some of the possible ways patients can become susceptible to mucormycosis include: human immunodeficiency virus/acquired immunodeficiency syndrome (HIV/AIDS), hematopoietic stem-cell transplant, lymphoid malignancies, neutropenia, hereditary immune deficiencies, immunosuppressive medications, diabetes mellitus, intravenous drug abuse and mechanical breakdown of the blood brain barrier through surgery or trauma [1]. The patient lacked the most common risk factors for this presentation including Diabetes Mellitus and immunosuppression. This patient did, however, have a social history risk factor of IV drug use and an environmental risk factor through his work in pest and termite control. Although these two risk factors are present, his source of infection is not clear. The patient lacked rhino-orbital and sinus involvement and exhibited no evidence of any cutaneous manifestations of disease. This patient had an indolent case of isolated intracerebral Mucormycosis with an unknown source of entry. This case highlights a less common path of illness, defined by isolated neurological deficits and an intracerebral brain lesion, which starkly contrasts the classic presentation of this infection.

In 2015, a retrospective clinical study was done by Jinjian *et al*. [2] that examined 81 cases of mucormycosis worldwide. The age range of patient cases was from 15 days to 79 years old with 41.6 mean years. According to their data, the only risk factor for this patient was that he was in his fourth decade of life, which was the most common decade of acquiring any mucormycosis infection. While it seems like the patient had a long hospital course, this is typical of intracranial mucormycosis infections reported in the literature. Moreover, Jinjian *et al*. [2] mentioned 11 different types of clinical classifications for mucormycosis presentation. This patient’s presentation according to their classification, can most accurately be described as Encephalitis mucormycosis due to the evidence of cerebral parenchymal damages, including hemiplegia, aphasia and evidence of increasing intracranial pressure. Four of the eighty-one cases reviewed by Jinjian *et al*. [2] presented with weakness and paralysis, which emphasizes the atypical nature of the patient presented here.

In 2016, Benachinmardi *et al*. [3] mentioned the significance of early identification of Mucormycosis infection due to the imperative nature of early treatment in determining patient outcomes. This case demonstrates the importance of this idea as it took 12 days from patient presentation to initiation of the Amphotericin treatment, which led to an unfortunate deterioration of the patient’s cognitive function. This was not the first case of late detection as seen in Rumbolt and Castillo [4], where a detailed course of an indolent case of intracranial mucormycosis is discussed. According to Jinjian *et al*. [2], 71.6 % of cases of mucormycosis are initially misdiagnosed. This figure is staggering but is one of which to be cognizant, due to the devastating nature of these infections.

### Conclusion

As evidenced by this case, isolated intracranial mucormycosis is a rare infection that can have an indolent course and must always be considered in a patient with a brain mass from an unknown source, even without evidence of paranasal sinus involvement. In addition, this case demonstrates the imperative nature of early identification of infection as misdiagnosis or mistreatment, may ultimately have fatal consequences. Lastly, this case highlights the importance of keeping this infection in the list of possible differential diagnoses for intracranial masses even in the absence of a clear entry point for infection. Although the patient described above has regained much of his neurologic functioning, the process has been slow requiring more than 3 months to regain the ability to speak. It is difficult to determine if a more rapid identification and treatment would have shortened this course but the patient’s hospital course demonstrates the importance of a timely diagnosis early in the disease course even if the patient does not have the ‘classic’ mucormycosis infection characterized by necrotic debris filling the nasal and sinus passages.
